# IgY antibodies: The promising potential to overcome antibiotic resistance

**DOI:** 10.3389/fimmu.2023.1065353

**Published:** 2023-01-20

**Authors:** Sherif A. El-Kafrawy, Aymn T. Abbas, Christopher Oelkrug, Marwa Tahoon, Sameera Ezzat, Alimuddin Zumla, Esam I. Azhar

**Affiliations:** ^1^ Special Infectious Agents Unit-BSL3, King Fahd Medical Research Center, King Abdulaziz University, Jeddah, Saudi Arabia; ^2^ Department of Medical Laboratory Sciences, Faculty of Applied Medical Sciences, King Abdulaziz University, Jeddah, Saudi Arabia; ^3^ Department of Clinical Pathology, National Liver Institute, Menoufia University, Shebin El-Kom, Egypt; ^4^ Biotechnology Research Laboratories, Gastroenterology, Surgery Centre, Mansoura University, Mansoura, Egypt; ^5^ Oelkrug Enterprises UG (haftungsbeschränkt), Ascheberg, Germany; ^6^ Epidemiology and Preventive Medicine Department, National Liver Institute, Menoufia University, Shebin El-Kom, Egypt; ^7^ MARC for Medical Services and Scientific Research, 6th of October City, Giza, Egypt; ^8^ Department of Infection, Division of Infection and Immunity, Centre for Clinical Microbiology, University College London, London, United Kingdom; ^9^ National Institute for Health and Care Research (NIHR) Biomedical Research Centre, University College London Hospitals, London, United Kingdom

**Keywords:** IgY antibodies, Bacteria, AMR, antibiotics, passive immunization

## Abstract

Antibiotic resistant bacteria are a growing threat to global health security. Whilst the emergence of antimicrobial resistance (AMR) is a natural phenomenon, it is also driven by antibiotic exposure in health care, agriculture, and the environment. Antibiotic pressure and inappropriate use of antibiotics are important factors which drive resistance. Apart from their use to treat bacterial infections in humans, antibiotics also play an important role in animal husbandry. With limited antibiotic options, alternate strategies are required to overcome AMR. Passive immunization through oral, nasal and topical administration of egg yolk-derived IgY antibodies from immunized chickens were recently shown to be effective for treating bacterial infections in animals and humans. Immunization of chickens with specific antigens offers the possibility of creating specific antibodies targeting a wide range of antibiotic-resistant bacteria. In this review, we describe the growing global problem of antimicrobial resistance and highlight the promising potential of the use of egg yolk IgY antibodies for the treatment of bacterial infections, particularly those listed in the World Health Organization priority list.

## Introduction

AMR occurs when a microorganism is able to survive in the presence of antibiotics at a concentration that would normally inhibit their growth (1). Antimicrobial resistant (AMR) bacteria are able to survive and thrive through natural selection (2). Multidrug resistance (MDR) refers to bacteria that are resistant to at least three classes of antibiotics. It arises from the presence of resistance-associated genes in the bacterial genome (3). Currently, an estimated 700,000 fatalities are attributed to (AMR) per year. By 2050, AMR could lead to about 10 million deaths per year, as well as a 2% to 3.5% loss in the gross domestic product and global social costs of up to 100 trillion USD (4).

A primary issue with the introduction of a new antibiotic is whether antimicrobial resistance (AMR) to it will eventually emerge (5). Multidrug resistance has been detected among gram-positive pathogens, with penicillin-resistant *Streptococcus pneumoniae*, methicillin-resistant *Staphylococcus aureus*, and vancomycin-resistant *Enterococcus faecium*, being of particular concern (6). Among gram-negative bacteria, resistance to third-generation cephalosporins followed by fluoroquinolones, carbapenems, and currently colistin among Enterobacteriaceae poses a global threat that has resulted in a large increase in mortality and treatment costs and changed the guidelines for the treatment and control of infection (7).

The major driver of resistance evolution is the overuse of antibiotics fostered by factors such as inadequate regulations and misuse, lack of awareness about proper practices and consequent unjustified or unskilled use of antibiotics, the use of antibiotics as growth promotors in poultry and livestock, unrestricted access to antibiotics (8). Antimicrobial resistance is a natural phenomenon that occurs over time and is usually due to genetic changes in an organism. Antimicrobial-resistant organisms are found in humans and all their living environments (animals, plants, water and soil) and can spread from human to human or through zoonotic transmission from animal(or animal products) to humans (9).

Resistance may be caused by one or more of the following mechanisms (2). Enzymatic inactivation of the antimicrobial compound as the case with beta-lactamases (10). Reducing the antimicrobial effect by modifying the metabolic pathways alters bacterial cell walls making antimicrobial agents lose their binding ability to the bacterial target (11). Modifying the antimicrobial targets includes overamplifying the target or altering the permeability of the cell membrane by either decreased influx (porin loss) or increased efflux (efflux pumps) leading to a reduction in the accumulation of antimicrobial agents inside the cell (12). Another mechanism by which bacteria might develop antimicrobial resistance is by acquiring efflux pumps that extrude the antibacterial agent from the cell before reaching the target site (11). However, antimicrobial resistance may be intrinsic or acquired; it can develop through the mutation of existing genes (13, 14), or through the transfer of genes from other species or strains (15, 16).

Strategies employed to overcome AMR include reducing the extensive use of antimicrobials, collecting and analysis of data, avoiding the overuse of antimicrobials in farm animals, and developing novel treatment approaches (17, 18). The development of novel nanoscale antimicrobial agents/nanocomposites has been reported on different microorganisms (18). The limitations, and/or health risks associated with these nano-sized particles need to be taken into consideration (19).

Antimicrobial peptides (AMPs) have been widely tested in the fight against AMR bacterial infections (20, 21). However, overuse of AMPs may result in more resistant forms of bacteria resulting in deadly infections (11). Another potential alternative is bacteriophages; these are bacterial viruses that act as pathogens against bacteria. They are abile to specifically attack and kill only their host bacterial cells (22). Their limitations include the development of antibodies after repeated treatment, rapid inactivation of phages by the spleen, endotoxin contamination of the therapeutic phage preparations from bacterial debris, limited host range, regulation, and bacterial resistance to phages (11). Plant-based therapeutic agents evolved as a therapeutic alternative due to the emergence of AMR infections and the growth of scientific knowledge about herbal medicines as a promising complementary treatment (23).

Antibodies, mostly produced in mammals, provide a useful alternative in the treatment of bacterial infections either directly by targeting bacterial surfaces or indirectly by neutralizing bacterial toxins and the virulence factors that are responsible for infection (24, 25). However, several challenges face the production of IgG antibodies in mammals including the weak immune responses of the antigens used, the pain and distress caused to animals by immunization, blood sample collection, and ultimately sacrifice (26) and the cost of the production, poor shelf life, and the scale-up required for the large-scale production (24, 27).

The search for a more efficient and economical approach for the production of antibodies without the harm caused to the animals has led to a growing interest in egg yolk antibodies (IgY) (28). IgY is an isotype of immunoglobulin found in birds. Large-scale production of antigen-specific IgY can be obtained from eggs laid by immunizing hens with the specific antigens (27). Passive immunization using IgY is a promising alternative approach to combat the emergence of new and current drug-resistant pathogens (29) The yield of IgY antibodies produced in eggs is 18 times superior to the amount produced in rabbits (30) thus reducing the number of animals needed and pain caused by blood collection and sacrifice (31). An extra benefit that IgYs provide is their high content of sialic acid (32) reported to increase the half-life of the drug (33) leading to the increased shelf life of the IgY antibodies. IgY antibodies retain activity through different manufacturing steps and dried IgY batches can keep their biological activity over several years (34, 35).

IgY antibodies are reported as potent preventive and/or therapeutic agents against several viruses such as Influenza A (36, 37), Rotavirus (38), Dengue (39), Zika (40), Ebola (41) and as we reported previously against MERS-CoV (42, 43) and SARS-COV-2 (44). IgYs were also tested for their anti-parasitic activities against *Trypanosoma cruzi* (45, 46)*, Cryptosporidium parvum* (47)*, Eimeria* (48)*, and Candida albicans* as a fungal infection (49).

According to the WHO, there are 12 bacterial priority pathogens for which novel antibiotics are urgently needed (50). IgY antibodies showed activity against most of these pathogens. The aim of this review is to highlight the potential role that specific IgYs can play in the immunotherapeutic prevention and treatment of these antimicrobial-resistant pathogens.

## Safety of the IgY antibodies with the different route of administration

IgYs are safer than IgGs as they do not bind to human Fc receptors or fix mammalian complement components; hence they do not induce dangerous immune responses (51). Hakalehto et al., 2021 reported that IgY antibodies are one of the safest possible therapeutic agents (52). IgY consumed orally is Generally Recognized as Safe (GRAS) by the U.S. Food and Drug Administration (53). Additionally, IgY antibodies have been used orally to treat pulmonary *Pseudomonas aeruginosa*-infected patients with no negative complications for up to 10 years (54).

Topical applications of the IgY antibodies (55) were reported against *S. mutans* as a gel or powder in a rat dental caries model and showed inhibition of *S. mutans* (56, 57). Patients who used toothpaste containing anti-gingivitis IgY showed significant differences in bleeding on probing and gingival index (58, 59). Short-term (three weeks) use of IgY mouth spray resulted in a significant decrease in *S. mutans* in dental plaque, and low levels of *S. mutans* were detected for at least 5 weeks after withdrawal of IgY (60).

Nasal delivery is superior in many cases to systemic delivery due to its non-invasive nature, fast onset of action, and low side effects due to targeted delivery (61–63). Anti*-P. aeruginosa* IgY were reported to inhibit murine pneumonia when administered intranasally (64). The protective effect and safety of intranasally administered anti-SARS-CoV-2 IgY antibodies were confirmed in a mouse model, with no adverse effects observed (65). Other reports showed that the superaficial application of anti-SARS-CoV-2 IgY would not be expected to elicit antibody-dependent enhancement of infection due to its topical application (53).

Systemic administration of IgY has not been clinically evaluated. IgY-based antivenom was given parenterally and showed complete protection in animal models of lethal venomous bites and stings (66–68). Moreover, intraperitoneal administration of polyvalent-specific anti-Zika virus IgY in a mouse model did not induce antibody-dependent enhancement (ADE) and did not display any side effects (40). More in depth studies on the safety and efficacy of IgY systemic delivery are needed before clinical use (69).

## IgY antibodies as a candidat to overcome antibiotic resistance

The concept of passive immunization describes the administration of specific antibodies obtained from an immunized donor in a prophylactic or therapeutic setting. In general, passive immunization is a naturally occurring means of transferring immunity from a mother to an offspring, such as through the immunoglobulins contained in breastmilk in mammals and the transfer of IgY antibodies through the egg yolk in chickens (70). The concept of passive immunization has drawn increasing interest in the past several years owing to the increase in antibiotic-resistant pathogens. In general, the IgY technology can be used to develop highly specific antibodies against a vast variety of antigens including bacteria, viruses, and even bacterial enzymes such as beta-lactamase, which is able to inactivate antibiotics. These antibodies are seen as a novel approach to targeting antibiotic-resistant bacteria through passive immunization. The administration of developed and isolated IgYs can provide rapid protection against diseases that are currently unresponsive to antibiotic therapies, including among immunocompromised patients for whom conventional treatment or vaccinations are not effective. The commercial availability of reagents that are specifically designed and developed for use in egg yolk antibody isolation and characterization has increased the amount of research in this area, with the term IgY being internationally recognized in research and industry (71). One of the humane advantages of IgY use is the ability to extract the antibodies from the eggs and not from blood, which makes it more favorable for animal safety and care (72). In the 1990s, several reports investigated the various aspects of IgY technology and applications (73). A particular advantage of IgY-based diagnosis and treatment is that the phylogenetic distance between mammals and birds enables the generation of IgY antibodies against conserved mammalian or pathogen proteins (74). This phylogenetic distance results in no recognition of mammalian Fc receptors and does not trigger the mammalian complement activation *in vitro* or *in vivo* (75, 76). IgY provide an added environmental advantage by using nontoxic techniques for purification (77). Precipitation techniques using water dilution and low pH-induced precipitation, as well as polyethylene glycol-, dextran sulfate- and xanthan gum-induced precipitations (78) or NaCl extraction (79) have been employed for IgY purification.

## Advantages of avian IgY antibodies over mammalian IgG for passive immunization

IgY antibodies have a large number of advantages over mammalian IgG antibodies such as cost-effectiveness, the short time needed for preparation and production, the wide range of potential pathogen targets, convenience in handling and storage, and the high yield of the target IgYs (53, 80). A hen can be considered a small “factory” for antibody production, as one hen can produce more than 22.5 g of total IgY per year of which 2% to 10% is composed of target-specific antibodies (81). This quantity is the equivalent of the IgG antibody production of 4.3 rabbits over the course of a year; further, this large amount of IgY can be harvested without killing the hen (29). IgY is more resistant to proteolysis than its mammalian IgG counterparts (82), and it has been found to retain 40% of its activity after incubation with trypsin or chymotrypsin for 8 hours (83). Moreover, owing to the phylogenetic distance between birds and mammals, immunizing laying hens twice with the specific antigen is enough to produce a humoral immune response that leads to the transfer of large amounts of specific IgY antibodies to the eggs for several months (53). IgYs are safer than IgGs as they do not bind human Fc receptors or fix mammalian complement components; hence, they do not initiate potentially dangerous immune responses (51). Owing to the lack of a hinge region between the two “arms” of the antibody molecule, the IgY molecular structure is more rigid than that of IgGs and thus somewhat stronger (84, 85).

## Advantages of IgY compared with antibiotics

The use of polyclonal IgY against infectious diseases minimizes the risk of developing AMR. Since the antibodies are directed to various antigens of the same microorganism, this lowers the chance of developing resistance to all of these antigens at the same time because they require multiple genes for synthesis (86). Therefore, specific IgY antibodies are promising alternatives for use as antimicrobials in human and veterinary health to combat the emergence of resistant bacteria (28). The use of IgY is environmentally friendly and elicits no undesirable side effects, disease resistance, or toxic residues (87). IgY-based therapy does not cause disruption of the host flora because the treatments target specific disease-causing pathogens (88).

## Target and antigen identification

The choice of a specific antigenic target depends on the characteristics of the pathogen and the therapeutic strategy itself. For example, IgY antibodies can target different factors important for the survival of bacteria such as enzymes, toxins, colonization factors, flagella, and mucosal receptors (89, 90). The actual mode of action of passive immunization includes the agglutination of bacteria, inhibition of bacterial adhesion, suppression of virulence factors, toxin neutralization, opsonization and enzyme inactivation (81).

The antigenicity of specific targets that are used to immunize the chickens can be influenced by the immunogen itself, the type of adjuvant used for immunization, route of antigen delivery, frequency of administration, and general avian properties (breed, commensal bacterial footprint, age, egg lying capacity) (91).

**Figure 1:**
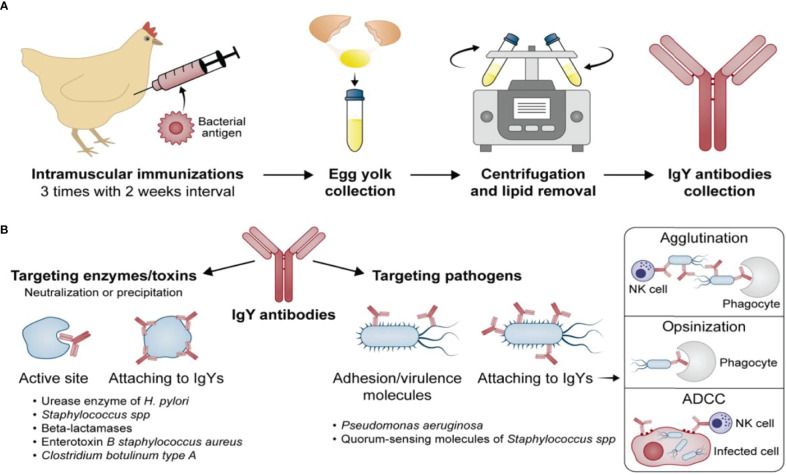
Development of the IgY antibodies **(A)** and their potential mechanisms of action **(B)**.

Four different target strategies of passive immunization with IgY antibodies can be distinguished and are further displayed in (**Figure 1**).

## Active site/whole enzyme

IgYs have been developed against crucial enzymes such as urease, which is expressed by bacteria such as *Helicobacter pylori* for survival in harsh conditions (low pH in the gastric mucosa), and beta-lactamases (92), which enable bacteria to degrade antibiotics and survive exposure to them. IgYs inhibit the enzymatic function (urease or beta-lactamase) through blockage of the active site, which prevents the substrate from binding to the enzyme and neutralizes the ability of bacteria to survive adverse conditions.

Research shows that IgYs generated against UreC, one of the subunits of urease, resulted in the prevention and even eradication of antibiotic-resistant *H. pylori* infections, which cause gastritis and gastric ulcers leading to gastric cancer (93). The addition of IgYs to yogurt as a functional food against *H. pylori*, for example, makes the usage of IgYs attractive (94).

Further research conducted by LeClaire et al. and Trott et al. (95, 96) also showed promising results regarding the therapeutic usage of IgYs generated against bacterial enzymes/toxins. These researchers reported that IgYs specific to enterotoxin B and botulinum type A neutralize their activity and can therefore prevent and treat infections due to *S. aureus* and *Clostridium* spp., respectively.

In addition, Hirai et al. (97) passively immunized mice with three different types of IgYs specific to *Vibrio cholerae* (anti-01, O139, and anti-cholera toxin B), which effectively prevented cholera infection in the mice.

## Adhesion molecules and virulence factors

Several studies have shown that it is possible to generate IgYs that specifically target pathogens directly. These IgYs act by blocking the adhesion/virulence molecules of the bacteria or by coating the whole bacterial cell wall, which suppresses the biological functions of the pathogen itself. This method was adapted by Nilsson et al. (98) who conducted a 12-year study on prophylactic oral IgY treatment against *Pseudomonas aeruginosa* in 17 patients and reported the prevention of bacterial colonization in most cases. These results clearly indicated the potential of IgYs as a preventative option for respiratory infections. IgY in this treatment is reported to form an antibody barrier preventing *P. aeruginosa* from entering the lungs through the nose/oropharynx and binding to the epithelial surface of the mucosa. Gargling with IgY at night keeps active concentrations of Anti-*Pseudomonas* IgY in saliva and oropharyngeal mucosa till the next morning (98).

Furthermore, Dapunt et al. (99) generated IgYs to target quorum-sensing molecules of *Staphylococcus* spp. associated with implant infections. In particular, the adhesion molecule AtlE (*S. epidermidis*), which is a member of the autolysin family and mediates the attachment to the implant surface, was identified as a target of interest. Autolysins themselves are a group of enzymes that catalyze the degradation of the bacterial cell wall at specific sites. Besides the quorum-sensing molecules, the bacterial heat shock protein GroEL was also investigated in the context of biofilm formation. GroEL is a highly conserved protein that is homologous to the human heat shock protein 60 (HSP-60) and is essential for protein folding. It was previously shown that bacteria are not able to survive without GroEL, which makes it a perfect candidate as a potential target. Immune cells are able to recognize GroEL, which induces several bactericidal strategies. So far, two scientific reports have mentioned GroEL in relation to IgY and as a potential target. Hermans et al. (100) focused on *Campylobacter jejuni* in broiler chickens and used whole cell lysate to immunize chickens for the generation of IgY antibodies specific against the pathogen. GroEL was identified *via* HPLC-MS/MS in the whole cell lysate, and it was predicted that the polyclonal IgYs would also contain an antibody portion against GroEL. Therefore, there is no clear indication of the impact of GroEL-specific IgYs on the growth of *C. jejuni*. Dapunt et al. (101) have also investigated GroEL-specific IgYs against *S. epidermis* in biofilm formation. Unfortunately, the impact was only minimal and further research is needed.

## Activity of the IgY antibodies against antibiotic-resistant bacteria

### Helicobacter pylori

*Helicobacter pylori*, a gram-negative, spiral-shaped, microaerophilic flagellated human bacterium. It colonizes the stomach causing acute and chronic gastritis developing peptic ulcers in 10% to 20% of cases, gastric adenocarcinoma in 1% to 2%, and gastric lymphoma in less than 1% of cases (102–105). *T*he misuse of antibiotics has increased resistance of *H. pylori* to CLR and MTZ which has reached 40%–50% and 70%–80% in some areas, respectively (106). As a consequence, antibiotic resistance has reduced the efficacy of standard triple therapy to 70% or lower (107, 108). Passive immunization with IgY directed against specific pathogens offers a potential alternative to antibiotics (109). Several *in vitro* and *in vivo* studies were performed to evaluate the efficacy of IgY antibodies against different *H. pylori* antigens including whole-cell bacteria (93, 110, 111), urease (89, 112–115), vacuolating cytotoxin A (VacA) proteins (116), neutrophil-activating protein (NAP) proteins (117), outer inflammatory protein (OipA) (118), cytotoxin-associated gene A (CagA) (119), and catalase (120). In addition, several *H. pylori* pathogenesis-related antigens can be used to generate IgY antibodies against different *H. pylori* targets such as sialic-acid-binding adhesion, blood group antigen binding adhesin, and γ-glutamyl transpeptidase (109). The mechanism of the IgY against *H. pylori* could be agglutination, help phagocytosis, or neutralization as well as blocking the adherence of the bacteria (109). Reports show that anti-*H. pylori* IgY antibodies effectively inhibit bacterial growth and adhesion to human gastric epithelial cells *in vitro*; also decrease *H. pylori*-induced gastric mucosal injury, improving gastritis, and attenuating gastric urease activity *in vivo* (109). Previously, we developed specific IgY antibodies from *H. pylori* cell lysate immunized chickens and evaluated their efficacy in a reliable *H. pylori*-infected mouse model with marked gastritis that was successfully developed by our research group (121). Results showed a significantly lower degree of infection and gastritis in IgY-treated animals than in untreated animals. A clinical trial was performed on 17 *H. pylori* asymptomatic volunteers who were orally administered egg powder containing anti-*H.pylori* urease IgY (Ovalgen, GHEN Corporation Inc., Gifu, Japan) for 4 weeks. The urea breath test (UBT) showed a significant decrease in UBT values although no case showed *H. pylori* eradication (122). Anti-*H. pylori* Urease IgY antibodies showed synergistic effects when taken at 3.4 g twice per day combined with lansoprazole (30 mg per day) for 8 weeks in a patient suffering from *H. pylori*-associated gastritis. The lansoprazole was taken to attenuate acid-induced inactivation of IgY. The treatment showed synergistic effects because IgY was shown to improve drug efficacy by reducing ulcer lesions (122).

Recently, a clinical trial evaluated multivalent IgY antibodies produced in chicken immunized with three recombinant *H. pylori* antigens urease B, flagellin A and antigen binding adhesion A2 (123). Ther trial included 94 *H. pylori*-positive volunteers, diagnosed as positive by a ^13^C UBT value of >4.0%. The recruited subjects were asked to administer a pack containing and 8.9 g of skim milk with 0.1 g of multivalent anti-*H. pylori* IgY 1 h before each meal for 2 weeks. The clinical symptoms of volunteers were followed up for 6 weeks after cessation of the administration period, followed by a ^13^C UBT which showed a decrease in UBT value by 56.0% with a total improvement rate of clinical symptoms in volunteers of 87.3%, and *H. pylori* eradication rate of 30.6% (123). The use of skim milk in this stusy was shown to effectively alleviate the degradation of IgY by pepsin under pH 1.2.

Horie K et al. (124) conducted a clinical trial on 42 volunteers divided into two groups. One group was given regular yogurt and the other was given yogurt mixed with 1.5 g of egg yolk containing about 45 mg of anti-*H. pylori* urease IgY three times daily. After oral administration of 2 and 4 weeks, the test group showed a significant reduction in the ^13^C UBT level by 34.19% and 39.3%, respectively with no side effects observed.

Several *H. pylori-*specific IgY antibodies have been applied for treatment; for example, a Chinese company used IgY antibodies as a raw material to develop chewing pills to treat and prevent *H. pylori* (125). Future advancements in antibody engineering will increase the application of IgY in passive immunization and therapy against *H. pylori* infections (125).

### Pseudomonas aeruginosa

*Pseudomonas aeruginosa* is a gram-negative opportunistic bacterium causing chronic respiratory infections in patients with cystic fibrosis, chronic obstructive pulmonary disease, as well as acute infections in immunocompromised patients (126). The excessive use of antibiotics for the treatment accelerates the development of *P. aeruginosa-*resistant strains, leading to the failure of the antibiotic treatment (127).

A preclinical evaluation of IgY against *P. aeruginosa* was reported in which the IgY binding to the bacteria was shown to prevent adhesion to the oropharynx and hence prevent bacterial colonization (128).

Anti*-Pseudomonas* IgY was shown to promote bacterial opsonization and augment the phagocytic activity of human polymorphonuclear neutrophils (PMNs) (129), and it was also found to induce bacterial clearance in an animal model (130). As IgY antibodies do not activate Fc-receptors, the observed IgY-enhanced PMN phagocytosis might not be triggered by the usual receptor-mediated engulfment of opsonized bacteria. Improved phagocytosis might due to recognition of IgY by receptors similar to avian IgY receptors (131) or non-receptor-mediated mechanisms such as alterations in physio-chemical environment of the bacteria, facilitating a more easy and rapid phagocytosis (132). In an animal model, IgY administration to the lung was reported to reduce bacterial burden 100-fold compared with controls, and it was also accompanied by diminished lung inflammation and reduced clinical symptom scores (133). A recent study in mice showed that IgY antibodies provided 100% protection against all strains of *P. aeruginosa* upon intranasal challenge with 2×10^7^ CFU directly into each nostril mixed with 500 μg of IgYs (133). Moreover, anti-flagellin IgY antibodies conferred protection against *P. aeruginosa* in a burned wound animal model, and they were found to confer dose-dependent efficacy covering all strain types (133). Sanches et al. (134) described an *in vitro* experiment showing the synergistic effects of IgY antibodies generated against SPM-1 or VIM-2-producing strains of *P. aeruginosa*. In that study, chickens were immunized with whole cell lysates of the bacterial strains, and the extracted IgY antibodies were tested in combination with the beta-lactam antibiotics ceftazidime, imipenem, or meropenem. The combination of IgYs and the beta-lactam antibiotics showed increased antimicrobial activity against resistant strains of bacteria. The authors were not able to describe the mode of action within this study.

Urinary tract infections (UTIs) with *P. aeruginosa* represent a major healthcare problem in disposed patients. *P. aeruginosa* establishes recalcitrant biofilm infections and can develop antibiotic resistance. In a recent study, *P. aeruginosa* (PAO1 and PAO3) was mixed with increasing concentrations of specific anti-*Pseudomonas IgY* (sIgY) or non-specific control IgY (cIgY) *in vitro*. The study showed a dose-dependent reduction in bacterial growth by the specific IgY at concentrations above 2.5%. *In vivo* effect of the IgY effect was evaluated in Balb/c mice which showed a reduction in vesical bacterial load by sIgY and cIgY when given the antibodies before infection (135).

A recent study evaluated the intranasal prophylactic effect of the anti-*P. aeruginosa* IgY antibodies (Pa-IgY) on the colonization of *P. aeruginosa* in the airways of a porcine model. Pa-IgY was administered through a nebulizer immediately before the administration of *P. aeruginosa*. A significant reduction was noticed in the Pa-IgY-treated group with the improvement of the physiological parameters (136). IgY against *P. aeruginosa* is now registered as an orphan drug in the European Medicines Agency (Designation number: EU/3/08/564). Patients were asked to gargle with IgY against *P. aeruginosa* in order to allow neutralization in the respiratory tract (137, 138). The clinical trial (NCT00633191) continued for about 10 years using a daily mouthwash containing 50 mg of specific IgY. None of the IgY-treated patients in this study became chronically colonized with *P. aeruginosa* with no side effects (54, 139). The activity of IgY against *P. aeruginosa* was shown in the saliva and oropharynx after gargle treatment with IgY solution (0.7 mg/ml) for 1 or 2 minutes and it was suggested that specific IgY is able to prevent *P. aeruginosa* invasion of the lungs (140). Phase III clinical evaluation (Clinicaltrials.gov Identifier: NCT01455675) of the effect of specific IgY antibodies on the recurrence of *P. aeruginosa* in the sputum of cyctic fibrosis patients asked to gargle with specific IgY solution every night after brushing their teeth. patients were followed up for 24 months or until the next *P. aeruginosa* infection whichever was first, no side effects were observed. Results showed a good tolerance profile for the immunoglobulin but did not show a clear therapeutic benefit of the *anti-P. aeruginosa* IgY treatment (141). The authors found that the placebo group reacted with far fewer events than expected, whereas the treated group followed the expected disease outcome. They hypothesized that the non-specific IgY may have unspecific inhibitory effects on protecting against reinfection of *P. aeruginosa* in cystic fibrosis patients.

### Salmonella

The *Salmonella* species, particularly *S.* Typhimurium and *S.* Enteritidis, are human and chicken pathogens (142).

In several studies, IgYs were generated against *S.* Typhimurium and *S.* Enteritidis and were found to exhibit significant agglutination (143, 144) and cross-reactivity (145), indicating the potential therapeutic effect of IgY generated against a specific *Salmonella* serovar infection for treating a broad range of different *Salmonella* strains.

Effectiveness of *Salmonella* treatment for *Salmonella* by specific IgY was shown to be more effective if protected from gastrointestinal degradation (146). The effect of encapsulation on the effect of IgY activity at low pH of the stomach was evaluated in birds against *Salmonella* Enterica ssp (SI) under *in vivo* conditions (**Table 1**). Birds were orally given 1 mL of bacterial suspension and then divided into three groups, group 1 was given *Salmonella* Immune powdered yolk (SIPY), group 2 was given *Salmonella* non-immune powdered and the third group was given *Salmonella* capsulated immune yolk (SCIY). For positive control, Enrofloxacin was added to the drinking water of a fourth group. A significant difference in the reduction of the colonization of SI, evaluated by cecal content, was found between the SCIY group on days 14 and 21 and the SA, SCIY treatments (147).

**Table 1:** Assessment of the efficacy of specific IgY antibodies against AMR for humans and animals.

Pathogen	Species	Dose and route	Response	References
*Helicobacter pylori*	Human	An anti-Hp Urease IgY-containing powder diet (Ovalgen, 900 mg), three times a day 30 min after each meal for 4 weeks.	Significant reduction in Urea breath test	(122)
	Human	Anti-HpU IgY (3.4 g/twice a day) for 8 weeks together with lansoprazole (30 mg per day)	Improving drug efficacy by reducing ulcer lesions	(122)
	Human	Administration of a pack (containing and 8.9 g of skim milk with 0.1 g of multivalent anti-H. pylori immunoglobulin Y	56.0% decrease in Urea breath test, 87.3% improvement in clinical symptoms and 30.6% *H. pylori* eradication rate	(123)
	Human	150 mL of drinking yogurt with 1.5g anti-IgY-urease 3 times daily	34.19% reduction in the urea breath test level by	(124)
*Pseudomonas aeruginosa*	Human	Daily mouthwash containing 50 mg specific IgY for about 10 years	Significant *P. aeruginosa* reduction with no adverse events	(54, 139)
	Human	Gargle with 70 ml containing 50 mg antipseudomonal IgY every night for 24 months	No significant difference between treatment and control IgY groups with good tolerance.	(141)
S*almonella enterica (SI)*	Chicks	Dietary supplemented 12.8 g/kg of Anti- IgY SI antibodies in capsulated form	Significant Salmonella colony count reduction in cecal content and liver tissue.	(147)
*salmonella Enteriditis* (SE) and *Salmonella Typhimurium* (ST)	Chicks	Lyophilized egg yolk containing IgY mixed with fed 5% (w/v) in capsulated or unencapsulated or mixed with probiotic 5% (w/v)	Significant reduction in colony count of Salmonella in the caecum	(148)
*E. coli and S. Typhimurium*	Pigs	Diet supplemented with a natural herbal additive containing IgY at concentrations of 0.5% or 1%.	Regulate the immune system and reduce the stress of microbial infections	(149)
*Avian pathogenic E. coli (APEC) O78 strain*	Chicken	100 mg specific IgY intramuscularly	Passive protection was achieved (90 -100%) from homologues challenge	(150)
*E. coli K88*	Pigs	Diet supplemented with yolk powder 400 mg/kg before infection	Complete reduction of E. coli 72 h post challenge	(151)
Enterotoxigenic *E. coli*, rotavirus, *salmonella*, and Shiga toxin-positive *E. coli*	Children	PTM202 (sachet containing 7 g of dried bovine colostrum and Dried whole egg) reconstituted in 30 mL one time per day for 3 days for a total of three sachets.	Reduction of diarrhea duration among children diagnosed to have one or more targeted organisms in their diarrheal stool	(152)
*C. jejuni* (Whole-cell lysate or hydrophobic protein)	Chicks	Egg yolk 5% (W/W) in feed 10 days before or after infection	Reduction of cecal jejuni count	(153, 154)
*Staphylococcus aureus*	Bovine	Intramammary infusion of 20 mg/mL IgY every 12 hours for 6 days	Reduced mastitis	(155)

In another trial, lyophilized egg yolk containing IgY from hens immunized either by *Salmonella* Enteritidis *(SE) or Salmonella* Typhimurium (ST) was orally delivered to a day old chicks mixed with their fed (Egg yolk only or encapsulated with liposomes 5% (w/w) or mixed with probiotic 5% (w/v) (**Table 1**). On day four, all chicks were challenged with (SE) or (ST) by oral inculcation using a blunt needle. One chick from each group was slaughtered on day 7th,14th, 21st and 28th. *Salmonella* was enumerated in ceacal content using SyBr Green real-time PCR. They found that serotype (SE, ST) specific anti-*Salmonella* IgY administered orally to the chicks significantly reduced *Salmonella* count in the caecum and there is no significant difference between the effect of the egg yolk only or encapsulate or egg yolk mixed with probiotics (148). On the other hand, IgY antibodies can resist digestion in the gastrointestinal tract of calves, remaining biologically active (35, 156).

The potential benefit of using IgY anti-*Salmonella* antibodies was proved when used in combination with probiotics which decreased colonization and fecal shedding in market-aged, young broiler chicks challenged with *S*. Enteritidis (157). This indicates the potential use of IgY anti-*Salmonella* antibodies in treating animal infections. *In vitro* evaluation of anti-*Salmonella* IgY antibodies in human epithelial Caco2 cells model showed that they prevent adhesion to cells (158).

An interesting study found that specific IgY against *Salmonella* could modulate the mucosal immune system of infected mice. Anti-*S.* Typhimurium IgY antibodies were orally administered with 0.4 mL of a solution containing 20 mg/kg once a day for 7 consecutive days after 3 days from infection. Nonspecific IgY or specific IgY has reduced the damage caused by *S.* Typhimurium challenge, and specific IgY treatment reduced jejunum ulceration, transmural inflammation, and edema significantly better than nonspecific IgY. Specific IgY diminished the effects of *S.* Typhimurium on the numbers of total T lymphocytes and CD8+ T cells while nonspecific IgY did not have the same effect (159). A commercial product is available in the market that contains IgY antibodies against *E. coli* and *Salmonella* (146).

IgY antibodies seem to be protective even if they are nonspecific. A study was performed on pigs infected with *E. coli* and *S.* Typhimurium that were fed a diet supplemented with a yolk sac containing 0.5% or 1% of IgY. Results showed oral egg yolk intake has regulated the immune system and reduced the stress due to microbial infections (149).

### Escherichia coli

*Escherichia coli* is a component of the intestinal microbiota with several pathotypes involved in the development of enteric and extraintestinal infections such as sepsis, diarrhea, urinary tract infections, and meningitis (160).

Studies in animal and laboratory settings suggest that IgY targeting animal enteropathogens is effective in the prevention and treatment of diarrheal symptoms (161). Hens may be simultaneously immunized with multiple antigens, resulting in polyvalent IgY antibodies targeting multiple steps of the pathogenesis process (162). Accordingly, the effect of using IgY antibodies as prophylaxis of diarrheal illness caused by enteric pathogens was evaluated *in vivo*. This IgY has strong inhibitory effects on enterotoxigenic *E. coli* (ETEC) adherence which is a critical first step in host colonization and subsequent toxin delivery (163). In the veterinary setting, IgY antibodies generated by immunizing hens with selected antigens from *E. coli* were evaluated for the ability to protect broiler chickens from diseases caused by avian pathogenic *E. coli* (APEC). Intramuscular IgY (100 mg) injection into broiler chickens followed by challenge with homologous (O78) *E. coli* through the intra-air sac route 3 days later which resulted in prophylaxis against *E. coli*-associated respiratory, enteric, and septicemic diseases (150) (**Table 1**). Another study showed that pigs that administered diets supplemented with 400 mg/kg of IgY targeting *E. coli* K88 strain before infection were recovered (diarrhea score=0) after 72 h of challenge compare to those treated with non-specific IgY (151) (**Table 1**). Anti-*E. coli* O111 IgY antibodies were also found to inhibit the growth of the target pathogens and five other mastitis-causing strains of *E. coli* (164, 165).

A proprietary mixture of dried bovine colostrum and dried whole egg (PTM202, PanTheryx, Inc., Boulder, CO, USA) was given orally to 301 Guatemalan children with acute non-bloody diarrhea in a randomized, double-blind placebo-controlled trial. The treatment was designed to target enterotoxigenic *E. coli*, rotavirus, *salmonella*, and Shiga toxin-positive *E. coli* (152) (**Table 1**). The PTM202 treatment given orally as one full reconstituted sachet once a day for 3 days, resulted in the reduction of diarrhea duration among children diagnosed to have one or more targeted organisms in their diarrheal stool at enrollment with no adverse events. The study concluded that this IgY-based treatment represents a potential alternative to treat acute diarrheal disease in low/middle-income communities.

### Campylobacter jejuni

*Campylobacter* species, particularly *Campylobacter jejuni*, is the most common etiology of human gastroenteritis worldwide (166). *C. jejuni* is transmitted to humans through poultry products with no effective eradication strategy from poultry production. Whole-cell lysate of *C. jejuni* was used to immunize chicken and the resulting egg yolk antibodies were fed to 6 days old chicks 5% (wt/wt) (**Table 1**). The chicks were inoculated orally with *C. jejuni* strain. Results showed that overall cecal *C. jejuni* count in chicken treated with *C. jejuni* IgY was significantly lower than the chicken treated with the nonspecific IgY antibodies. In addition, transmission to contact chicks was completely prevented (153).

In another recent study (154), two novel vaccines, a bacterin of 13 C*. jejuni* and *C. coli* strains and a subunit vaccine of six immunodominant *Campylobacter* antigens, were injected to immunize laying hens producing prolonged high levels of specific IgY in egg yolks. *In vivo* trial, yolks were orally in broiler feed 5% (wt/wt) for prophylaxis (11 days before infection) resulting in significant reduction in the number of *Campylobacter*-colonized broilers. In the therapeutic arm of the *in vivo* trial, administration of the IgY for 3 days mixed with fed 5% (wt/wt) resulted in a significant decrease in *C. jejuni* counts per infected bird. The hyperimmune yolks showed strong reactivity to a broad spectrum of *C. jejuni* and *C. coli* indicating that this passive immunization approach offers possibilities to control *Campylobacter* colonization in poultry (154).

### Acinetobacter baumannii

*Acinetobacter baumannii* is a gram-negative bacillus that is a common cause of nosocomial infections. It is responsible for hospital-acquired sepsis, ventilator-associated pneumonia, skin and soft tissue infections, wound infections, urinary tract infections, secondary meningitis, and bloodstream infections (167, 168). Nosocomial outbreaks of *A. baumannii* present a considerable threat to ICU patients and are associated with increased mortality, longer hospital stays, and higher treatment costs (169). The wide use of broad-spectrum antibiotics has caused most *A. baumannii* strains to develop resistance to multiple antimicrobial agents (170), rendering the bacterial infection difficult to cure (171, 172).

*In vitro* study indicated that specific IgYs inhibited the growth of pan-drug-resistant *A. baumannii* (PDR-Ab) in a dose-dependent manner. The antimicrobial efficacy of the two IgYs developed against two *A. baumannii* strains were comparable to that of cefoperazone/sulbactam. Both IgYs showed significant growth inhibition of PDR-Ab at 20 mg/mL within 24 h (173).

Specific IgYs were reported to enhance bacterial agglutination, causing a CFU reduction rather than directly affecting individual bacteria (173). The binding of IgY to the bacteria was shown to cause cell crenation and structural modification on the cell surface, resulting in reduced bacterial attachment to the mucosa. The same effect was shown for specific IgY against *H. pylori* attaching to gastric cancer cells (93), and *Salmonella* attaching to intestinal cells (174, 175).

OmpA and Omp34 are essential virulence factors involved in *A. baumannii* adhesion to the human lung epithelial cell line. The protective effect of specific anti-acinetobacter IgYs raised against OmpA, Omp34 and inactivation of the whole-cell of *A. baumannii* was demonstrated (176). The therapeutic activity against the same antigens (OmpA and Omp34 or inactivated whole-cell of *A. baumannii)* was also conducted in another study, in which BALB/c mice were intranasally administrated 1.18 × l0^6^ to 6 × l0^8^ CFU *A. baumannii*, after 4 hours, 40 or 100 μg of specific IgY antibodies were intranasally administrated with therapeutic effect in a murine pneumonia model (177). *A. baumannii* increases its antimicrobial resistance through biofilms formation (178). Intranasal administration of anti- biofilm-associated protein (Bap) IgY antibodies was found to inhibit antibiotic-resistant strains of *A. baumannii* through the inhibition of biofilm formation (179–181).

Another *in vivo* study showed that intraperitoneal injection of anti-*A. baumannii* IgY antibodies in nasally infected BALB/c mice inhibited bacterial growth and protected mice from acute pneumonia induced by *A. baumannii*, suggesting the potential of these specific IgYs to be used as a new therapeutic alternative to treat PDR-Ab infections in humans (182).

### Mycobacterium tuberculosis

Treatment of drug-resistant *M. tuberculosis* is a major health concern because such cases require second-line antibiotics, which are less effective, more expensive, and more toxic (183). Immunotherapy might provide an alternative for the treatment of drug-resistant TB strains, with promising outcomes and better quality of life for patients (184). In a rat peripheral blood mononuclear cell model, administration of high concentrations of IgY anti-M bacterium tuberculosis (anti-MBTC) increased interleukin (IL)-2 and interferon (IFN) expression (185). Production of these components has a major role in controlling antibody- and cell-mediated immunity, and the study results showed that IgY anti-*M. tuberculosis* could increase the production of IL-2 and IFN-γ and the proliferation of rat peripheral blood mononuclear cells in a concentration-dependent manner. Hens were immunized with four 80-μg doses of antigen, and anti-MBTC IgY antibodies in eggs were reported to reach a peak concentration at 4 weeks after immunization and to persist for 200 days after immunization. Western blot analysis showed the presence of anti‐MBTC IgY in egg yolks, with molecular weights of approximately 78 kDa (184). The authors concluded that IgY against MBTC may warrant evaluation for use in combination with other immunotherapeutic treatments of tuberculosis.

### Staphylococcus aureus

The economic burden of antibiotic-resistant *S. aureus* (e.g., methicillin-resistant *S. aureus*) infections affects not only individual patients but also the healthcare systems of different countries owing to the persistence of infection, recurrent infections, a wide spectrum of clinical presentations, and diminished quality of life (186). Passive immunotherapy might provide an alternative for high-risk patients with prolonged hospitalization (187); however, native cross-species antibodies induce violent immune reactions. Additionally, *S. aureus* immune defenses, such as staphylococcal protein A (SpA) and staphylococcal binding immunoglobulin (Sbi), bind the Fc portion of these antibodies in reverse orientation to avoid complement-mediated killing and phagocytosis. IgY antibodies targeting SpA were tested *in vitro* and found to completely inhibit the growth of *S. aureus* at a concentration of 150 μg/mL, and to inhibit biofilm formation by ~45% showing potential use to neutralize these infections (188).

Specific IgY antibodies generated against *S. aureus* were shown to reduce mastitis during a 6-day intramammary infusion of 100 mg/mL IgY twice a day (155) (**Table 1**). Specific IgY against encapsulated type 5 (IgY-T5) and type 8 (IgY-T8) and non-encapsulated type 336 (IgY-T336) *S. aureus* strains (at 5 mg/mL) significantly blocked the internalization of bacteria bovine mammary epithelial cells within 6 h (189). authors suggest that the generated IgY antibodies control mastitis by preventing the uptake rather than by inhibiting the growth of bacteria.

## IgY monoclonal antibodies

Successful generation of the monoclonal IgY or IgY fragments in the last few years has increased the functional use of IgY fragments, such as single chain (scFv) (190), chimeric (191), and humanized IgY (192). Monoclonal IgY antibodies combines the benefits of avian IgY antibodies and the features of monoclonal antibodies (193). They have the potential for use as therapeutics in both veterinary and human applications, immunological detection and diagnosis, and for screening and validating biomarkers (193).

Phage display production of IgY monoclonal antibody is more likely to generate a robust immune response against various highly conserved mammalian protein molecules (194). Monoclonal IgY antibodies conjugated with phthalocyanine—a synthetic photosensitizing dye used in near-infrared phototherapy—were recently used against *Candida albicans* and provided highly effective and specific success in an *in vivo* skin infection model with no damage to the healthy epithelium (195). In another study, hens were immunized with canine parvovirus VP2 (CPV-VP2) virus-like particles (VLP) and the specific IgY-scFv were generated using the T7 phage display technique (196). Transgenic IgY antibodies containing the bird constant regions and the human variable regions allow the use of the highly specific IgY antibodies against mammalian conserved proteins (197). So far one clinical trial is reported on the parenteral administration of monoclonal IgY product Sym021 (trial ID: NCT03311412) against human programmed cell death protein 1 (PD1) with promising inhibitory binding to the target protein (198).

## Marketing of the IgY antibodies for immunotherapy

The vast number of research studies in the past few years has led to an increase in the number of registered products for therapy and diagnosis together with an increase in the number of patents filed and clinical trials registered. For human use several IgY products are registered in the market such as IgY Max (against 26 human-relevant bacteria), Ig-Guard Helico, GastimunHP, and Ovalgen^®^ HP (*Helicobacter pylori*) (69). For veterinary use, about 56 products are reported at various stages of evaluation, including products in the market such as Ig-Guard Calf, Ig Lock Calves, Globigen^®^ Dia Stop, and IgY DNT (for calf diarrhea); PG-002 (for cow mastitis); Ig-Guard Swine, Ig Lock Pig (for swine diarrhea), Ig-Guard Puppy, ParvoONE^®^, Ig Lock Canine, GastroMate^®^, and Guardizen (for pets, especially in canines); Ig-Guard Duck, Ig-Guard Poultry, BIOAb DHV-IgY (for poultry) (69)

The market value of IgY polyclonal antibodies is estimated to be USD 14.2 million by 2027. However, the total antibody market share is only 0.24% (199). About 95% of IgY antibody productions are polyclonal antibodies with future prospects of IgY fragments and monoclonal IgYs (69). Several of the production companies now available provide custom antibody production services (53, 200). As the concept of using IgY antibodies in therapy is a new approach for alternative treatments, the USFDA has enforced strict regulations for the parenteral administration products (193, 201) which mandates more studies on the safety and efficacy of this route of administration. The documentation in these studies is an essential part of the process as well as the utility of standard processes such as good manufacturing practice (GMP) conditions, and immunization of specific- pathogen-free (SPF) birds (69).

## Limitations of the IgY antibodies

The susceptibility of IgY to proteolysis is one of the limitations to the oral use of IgY for passive immunotherapy. Although IgY antibodies are resistant to inactivation by the gastric proteolytic enzymes trypsin and chymotrypsin, it is degraded by pepsin (82). To overcome this obstacle, microencapsulation is found to be an effective approach to protect IgY from gastric inactivation (202).

The lack of standardization in the experimental animals (i.e., specific-pathogen-free birds) for the production and extraction and purification procedures of IgY antibodies is one of the major difficulties facing the progress in product licensing so far as well as the consensus on regulation and approval of IgY-based health products (53). More safety studies are needed to evaluate their safety for use as human and veterinary therapeutics. Research is also needed to develop more industrial scale standardized extraction and purification methods to fit the needs of clinical applications (69).

## Conclusion and future prospective

MDR pathogens are a growing threat to human health and welfare. The problem requires more research into innovative, and effective approaches including immunotherapies. Economically one of the main advantages of avian immunoglobulins is their cost-effective production, with the benefit of upscaling in the poultry industry, which may allow low-income countries to easily adopt technological capacities in their health systems. Overall, the use and application of IgY antibodies will emerge as an alternative to antibiotics and will help in the design of novel, safe and effective biologicals for the treatment of various MDR pathogens.

## Author contributions

SE-K, Conceptualization, Supervision, Writing – original draft, Writing – review & editing; AA, Conceptualization, Supervision, Writing – original draft, Writing – review & editing. CO, Conceptualization, Supervision, Writing – original draft, Writing – review & editing. MT, Writing, original draft, Writing – review & editing. SE Writing, original draft, Writing – review & editing. AZ, Validation, Supervision, Writing – original draft, Writing – review & editing. EA, Conceptualization, Supervision, Writing – original draft, Writing – review & editing. All authors contributed to the article and approved the submitted version.
